# Simultaneous UV/vis Absorption in Parallel Configuration, Photoluminescence and Raman Spectroelectrochemistry

**DOI:** 10.1021/acselectrochem.5c00038

**Published:** 2025-03-22

**Authors:** Fabiola Olmo, Martin Perez-Estebanez, Aranzazu Heras, Francisco Javier del Campo, Alvaro Colina

**Affiliations:** † Department of Chemistry, 16725 Universidad de Burgos, Pza. Misael Bañuelos s/n, E-09001, Burgos, Spain; ‡ BCMaterials, Basque Center for Materials, Applications and Nanostructures, UPV/EHU Science Park, 48940 Leioa, Vizcaya, Spain; § IKERBASQUE, Basque Foundation for Science, 48009 Bilbao, Spain

**Keywords:** spectroelectrochemistry, UV/vis absorption, photoluminescence, Raman, EC-SERS

## Abstract

Analytical Chemistry is the science of chemical measurements that seeks to acquire the most comprehensive information about a chemical system. Recent advances in technology have facilitated the development of new combined analytical techniques capable of supplying analytical signals of different natures. These signals subsequently provide diverse information related to specific chemical reactions. This technical note proposes a new combination of three different spectroscopic techniques (UV/vis absorption spectroscopy in a parallel configuration, photoluminescence and Raman spectroscopy) with electrochemistry. To illustrate the capabilities of this new technique, two chemical systems (tris­(2,2′ bipyridine)­ruthenium­(II) and ofloxacin) were selected. A comparison of the behavior of the two molecules during the electrode process demonstrates the advantages of obtaining several signals simultaneously in a single experiment.

## Graphical abstract

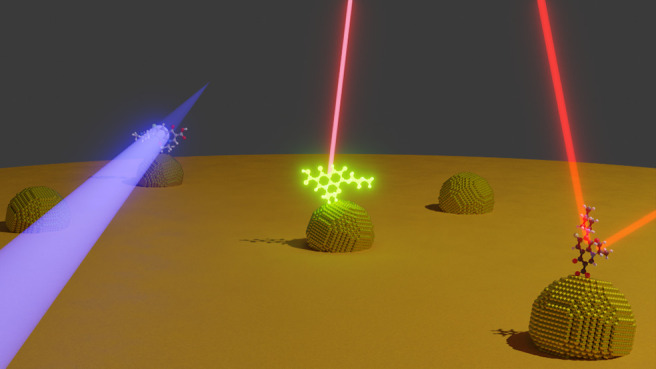

## Introduction

Spectroelectrochemistry (SEC) provides a great amount of information in a single experiment, since it permits the following of a system from two different points of view: electrochemical and spectroscopic.^1^ To enhance the capabilities of electrochemical analysis, we propose to implement new multiresponse techniques that enable the simultaneous recording of multiple signals.^2,3^ Several SEC devices combining two spectroscopic techniques have been developed by our group in the past, promoting the development of new SEC techniques such as bidimensional UV/vis absorption SEC,^3,4^ the combination of fluorescence and UV/vis absorption SEC,^5^ or the combination of Raman scattering and UV/vis absorption SEC.^6^ Nevertheless, to the best of our knowledge, this is the first time that electrochemistry is coupled to three different spectroscopic techniques simultaneously.

In this technical note, we propose a novel design that allows the straightforward and reproducible coupling of *operando* and simultaneous SEC measurements of UV/vis absorption spectroscopy, Raman scattering, and photoluminescence (PL). This new setup focuses on the wealth of information that all these techniques can provide in the study of a chemical system. UV/vis SEC provides molecular information about the compounds present or electrogenerated at the electrode/solution interface and in the diffusion layer. PL SEC also allows the acquisition of data regarding the location and characteristics of molecules at the electrode/solution interface and within the diffusion layer, achieving a higher degree of specificity and sensitivity than UV/vis absorption SEC, as these molecules are fluorescent. Finally, Raman SEC provides vibrational characterization of the compounds, being surface-sensitive due to the *Surface Enhanced Raman Scattering* (SERS) phenomenon.

To characterize complex systems, such as electrode processes,^7,8^ SEC experiments can be performed separately, changing the optical technique used in each case. Nevertheless, our proposal is that the simultaneous combination of different analytical techniques represents a better strategy, as it facilitates the study of the same chemical system under the same experimental conditions, thereby avoiding any influence of irreproducibility among consecutive experiments. In some cases, the studied system can exhibit inherent reproducibility issues, making it difficult to establish reliable correlations between experimental results.

## Experimental Section

Instrumentation, reagents and materials, synthesis of gold nanoparticles (AuNPs), and electrode modification are described in the Supporting Information (SI).

### Design and Fabrication of the Device for UV–vis absorption/PL/Raman SEC Measurements

Figure 1 shows the multi-SEC cell for the simultaneous recording of UV/vis absorption, PL, Raman, and electrochemical signals. This cell consisted of three main parts: I) support of the screen-printed electrode (SPE), II) holder of the optical fibers for UV/vis absorption measurements in a parallel configuration, and III) holder of the optical fibers for PL measurements. A detailed description of the SEC cell is provided in the SI.

**Figure 1:**
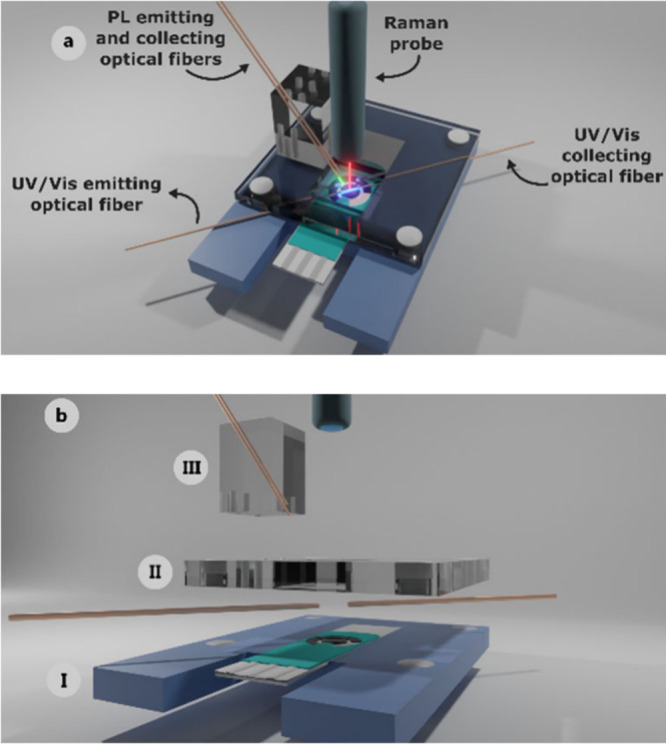
(a) Schematic of the assembled UV/vis absorption/PL/Raman SEC cell and (b) detailed schematic view of each part of the cell. (I) Lower piece, (II) Middle piece and (III) Upper piece.

## Results and Discussion

### Validation of the Cell

The SEC cell shown in Figure 1 and described in the SI will be validated by studying a typical fluorescent redox compound, tris­(2,2′-bipyridyl) ruthenium­(II) (Ru­(bpy)_3_^2+^). The behavior of the system under study can be observed by simultaneously performing electrochemistry and three spectroscopic techniques (UV/vis absorption, PL, and Raman spectroscopy). For this purpose, a 10^–3^ M Ru­(bpy)_3_^2+^ aqueous solution in 0.1 M KNO_3_ was studied. Ru­(bpy)_3_^2+^ undergoes a reversible oxidation–reduction process with the transfer of one proton and one electron.^9^ Several studies also report UV/vis absorption,^10^ PL^10^ and Raman spectra^11,12^ of the reduced and oxidized forms of this complex. Here, Ru­(bpy)_3_^2+^ was studied in a Au-SPE modified with drop-casted AuNPs (see SI). A cyclic voltammetry (CV) was performed between −0.10 V and +1.10 V at 0.02 Vs^–1^, starting at +0.60 V in the anodic direction. Simultaneously, the evolution of the UV/vis absorption spectra in the parallel configuration, the PL spectra and the Raman scattering spectra of the species consumed and generated at the electrode surface were recorded. All experiments were performed with an integration time of 1 s for the three optical techniques, although different integration times could be also used for each spectroscopic technique. It should be noted that the reference spectrum for UV/vis absorption was recorded at OCP. Therefore, the changes in absorbance during the experiment were measured.

The evolution of the spectra with potential/time is shown in Figure S3. The UV/vis absorption spectra (Figure S3a) are characterized by a positive absorption band at 312 nm and other negative band at 452 nm, which is accompanied by a small shoulder at 352 nm. Since the registered spectral changes are compared with the initial solution, it is possible to observe negative absorption bands corresponding to the disappearance of the Ru­(bpy)_3_^2+^ species and positive ones related to the generation of Ru­(bpy)_3_^3+^. In Figure S3a, the band that emerges at 312 nm is due to the electrogenerated Ru­(III) complex and is assigned to a ligand-centered π–π* transition (LC) in the heterocyclic ligand,^9,13^ while the negative band centered at 452 nm is related to Ru­(bpy)_3_^2+^ disappearance and is ascribed to a metal-to-ligand (d−π*) charge transfer (MLCT) process.^13^ The shoulder at 352 nm is attributed to the metal-centered (MC) d–d transition of Ru­(bpy)_3_^2+^. The evolution of the PL spectra (Figure S3b) represents the variation in fluorescence taken with respect to the initial fluorescence spectrum. As can be seen, it exhibits a band at 630 nm attributed to fluorescence emission from the MLCT triplet excited state (^3^MLCT) to the ground state of Ru­(bpy)_3_^2+^, as the evolution of photoluminescence spectra is negative during oxidation of these molecules.^9^ Regarding the evolution of Raman spectra (Figure S3c), several peaks can be observed, corresponding with the classical reported Ru­(bpy)_3_^2+^ SERS spectra.^14^ Table S1 summarizes the Raman band assignment for this experiment.

Figure 2 shows the cyclic voltammogram (CV) and the evolution of the most characteristic band for each spectroscopic signal with respect to the applied potential. The CV (Figure 2a) shows a reversible oxidation–reduction process and an irreversible process. The anodic peak (O_1_) that reaches its maximum around +0.98 V indicates the oxidation of the Ru­(bpy)_3_^2+^ to Ru­(bpy)_3_^3+^.^15^ During the backward sweep, two reduction peaks (R_1_ and R_2_) are observed. The first cathodic peak (R_1_, +0.90 V) is associated with the reduction of Ru­(bpy)_3_^3+^ to Ru­(bpy)_3_^2+^.^15^ A second cathodic peak (R_2_, +0.23 V) appears in the CV, which is related to the reduction of gold oxide generated on the NPs deposited on the WE surface.^16^ It can be deduced that during the oxidation of the Ru­(bpy)_3_^2+^ complex, also the oxidation of the AuNPs takes place, but the electrochemical signal does not provide direct evidence of this information. Replicating this experiment in the absence of AuNPs did not lead to a change in the CV.

**Figure 2:**
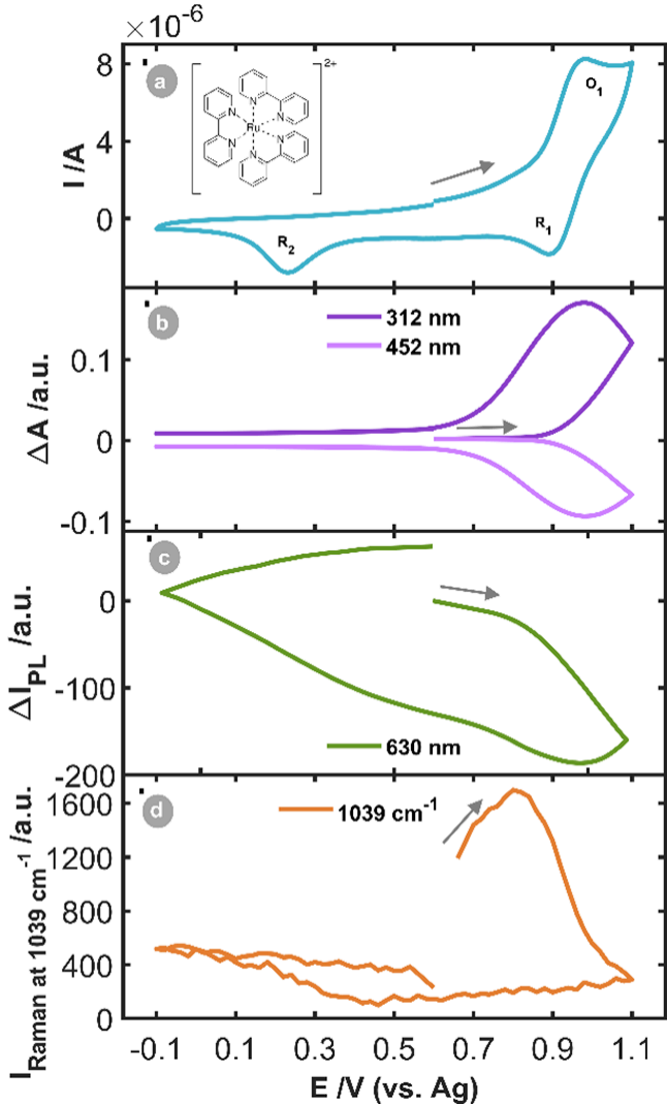
SEC experiment of 10^–3^ M Ru­(bpy)_3_^2+^ in 0.1 M KNO_3_ between the vertex potentials −0.10 V and +1.10 V, starting at +0.60 V in the anodic direction at 0.02 V·s^–1^. (a) CV and (b) CVA at 312 and 452 nm, (c) CVF at 630 nm and (c) CVR at 1039 cm^–1^.

Figure 2b shows the evolution of absorbance at 312 and 452 nm with potential, which is known as the cyclic voltabsorptogram (CVA). This representation reveals how from +0.85 V onwards absorbance at 312 nm increases until the system reaches a potential of +1.10 V during the backward scan, which matches with the oxidation-reduction of the coordination complex studied. The CVA at 452 nm displays the opposite behavior, decreasing the absorbance from +0.85 V onwards until the potential of +1.10 V in the backward scan is applied. This information is congruent with the reversible process observed in the CV (Figure 2a) which must be ascribed to the reversible oxidation of Ru­(bpy)_3_^2+^. The signal at 312 nm corresponds to electrogenerated Ru­(bpy)_3_^3+^ while that at 452 nm corresponds to consumed Ru­(bpy)_3_^2+^. UV/vis SEC does not provide information about the second reduction peak (R_2_) because, in the parallel configuration, only the diffusion layer is interrogated. PL-SEC also demonstrates that the redox process observed at +0.94 V is due to the reversible oxidation of Ru­(bpy)_3_^2+^. Figure 2c represents the cyclic voltafluorogram (CVF) at 630 nm (evolution of PL with the applied potential) supplying information about the same reversible redox process, as was observed with UV/vis SEC: first, the fluorescence decreases from +0.80 V onwards due to the oxidation process and the generation of Ru­(bpy)_3_^3+^ (O_1_); in the backward scan when the applied potential is cathodic enough to regenerate Ru­(bpy)_3_^2+^ the fluorescence increases recovering the starting value (R_1_).^9^ In the cyclic voltaRamangram (CVR) at 1039 cm^–1^ (evolution of Raman intensity with the applied potential, Figure 2d) Raman intensity increases until +0.80 V in the onward scan, related to the interaction of Ru­(bpy)_3_^2+^ with the AuNPs on the WE surface. The decay of the Raman intensity from +0.80 V onwards coincides with the oxidation onset potential shown in the CV (O_1_), due to the oxidation of both, AuNPs and Ru­(bpy)_3_^2+^. This Raman intensity decreases until the gold oxide generated on the surface is reduced (R_2_) (around +0.40 V), where a slight increase in the signal is observed due to the regeneration of the SERS substrate. This behavior can be confirmed by following a typical Raman band of gold oxides peaking at 570 cm^–1^ (Figure S4a) where an increase of Raman signal at 570 cm^–1^ is observed from +0.60 V and +0.35 V in the backward scan, when the regeneration of the SERS substrate occurs (Figure S4b, blue line). These results confirm that AuNPs are oxidized concomitantly with the oxidation of the molecule.

This validation experiment shows how the three spectroscopic signals can be registered simultaneously, and that all of them are related to the same redox system, the redox pair Ru­(bpy)_3_^3+^/Ru­(bpy)_3_^2+^ supplying all kinds of information related to the charge transfer process, as well as the correct synchronization between the electrochemical and spectroscopic signals in this multi-SEC cell.

### Application of the Cell to Study a Bactericidal Molecule

Experiments with Ru­(bpy)_3_^2+^ allowed us to demonstrate that simultaneous information from four different points of view can be obtained with the cell proposed in this work. As proof of concept, the oxidation of a fluorescent organic molecule, ofloxacin (OFL), a commonly used antibiotic, is proposed. This system was selected because its spectroscopic behavior is similar to Ru­(bpy)_3_^2+^, but its Raman response is quite different. In addition, the study of this molecule is of great interest because of its widespread use as a bactericidal agent. OFL exhibits characteristic UV/vis absorption, fluorescence emission, and Raman spectra that change when subjected to electrochemical oxidation processes. The performance of time-resolved experiments in which the evolution of the electrochemical signal, molecular absorption, fluorescence emission and Raman spectra are recorded simultaneously will be of great help in determining its reaction mechanism, which may help to understand its mode of action as a bactericidal antibiotic.^17,18^

For this purpose, a 5 × 10^–4^ M OFL solution in Britton-Robinson buffer (BR, pH = 4.25) was prepared to perform SEC experiments while maintaining the above experimental conditions. Similarly, CV was selected as the electrochemical technique. In this case, changes were recorded between the vertex potentials +0.00 V and +1.10 V, starting at +0.60 V in the cathodic direction. Two scans were registered at 0.02 V·s^–1^, and the initial spectrum at OCP was selected as reference for UV/vis absorption measurements. All spectra obtained using the three techniques were recorded at an integration time of 1 s. However, a different integration time could be used for each individual technique, depending on the signal to noise ratio.

Figure S5 shows the contour plots for each spectroscopic technique registered during the SEC experiment for the oxidation of OFL. In this case, the evolution of UV/vis absorption spectra (Figure S5a) is characterized by a strong negative absorption band at 303 nm which corresponds to a π–π* electronic transition of the chromophore of OFL that involves a carboxyl group and the nitrogen atom at position 1 in the quinolone group, and a broad negative shoulder between 330 and 370 nm, linked to an n−π* electronic transition of the chromophore of OFL from the nitrogen of the piperazinyl group to the carbon at position 7 in the quinolone group attached to the carbonyl group; both bands are related to the consumption of OFL on the WE surface due to its oxidation.^2^ The evolution of PL spectra (Figure S5b) shows a band at 521 nm that decreases during the oxidation of OFL that it is accompanied by a small shoulder between 390 and 460 nm that increased during this oxidation process.^19^ The emission band peaking at 521 nm is related to the protonated form of OFL, indicating again the consumption of this molecule by the oxidation process. The increase in the shoulder at shorter wavelengths must be linked with the oxidation product electrogenerated.^20^ To facilitate a more accurate visual representation of the photoluminescence signal’s evolution, Figure S5b illustrates its variation throughout the experiment, taking the initial fluorescence spectrum of the solution as a reference spectrum (ΔI_PL_ = I_PL,t_ – I_0_, where I_PL,t_ is the photoluminescence intensity at different times/potentials applied during the experiment, and I_0_ corresponds to the photoluminescence intensity of the initial spectrum of the solution). Finally, the evolution of the Raman spectra (Figure S5c) is dominated by two peaks at 1397 and 1619 cm^–1^ that evolve during the second potential cycle. The first one is due to the symmetric stretching mode of the carboxylate group (ν_ring_, COO^–^), and the second characteristic band is attributed to the C=C stretching vibration of the quinolone aromatic ring (ν_s_, C=C).^21,22^

Figure 3a shows the corresponding CV. No reduction peak was observed during the first cathodic scan. When the potential is reversed in the anodic direction, a first oxidation peak (O_1_) at +0.95 V is observed due to the oxidation of the AuNPs and OFL. OFL is irreversibly oxidized, as was demonstrated in previous works.^2^ Regarding the second cycle, a cathodic peak (R_2_) is observed at +0.28 V, corresponding to the reduction of AuNPs, oxidized at potentials higher than +0.75 V, which agrees with the experiments with Ru­(bpy)_3_^2+^.

**Figure 3:**
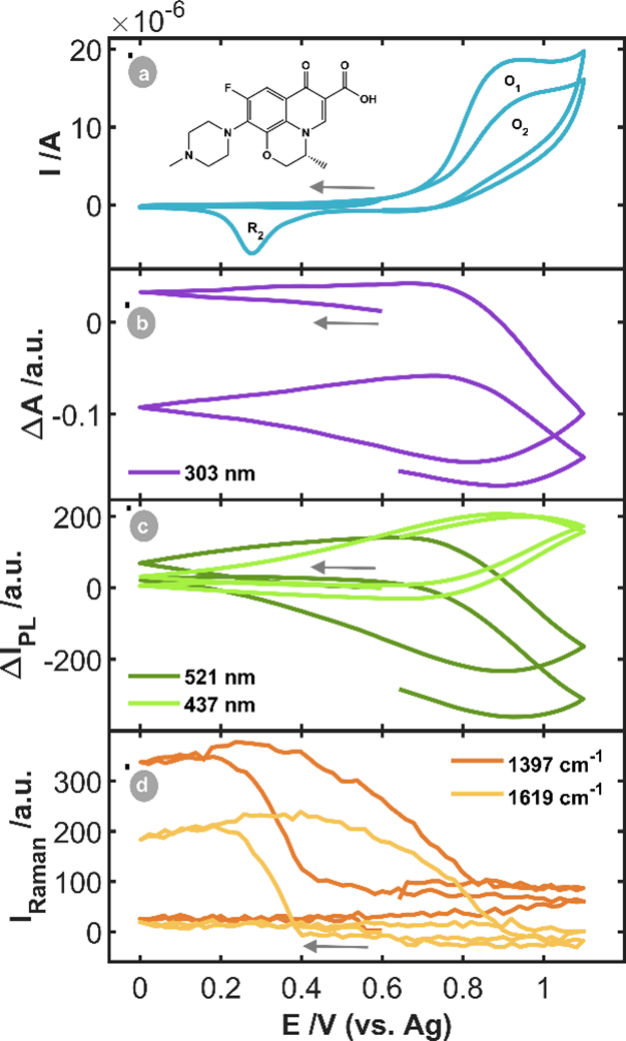
SEC experiment of 5·10^–4^ M OFL in BR pH = 4.25 between the vertex potentials +0.00 V and +1.10 V, starting at +0.60 V in the cathodic direction at 0.02 V·s^–1^. (a) CV and (b) CVA at 303 nm, (c) CVF at 521 and 437 nm and (c) CVR at 1397 and 1619 cm^–1^.

The CVA at 303 nm (Figure 3b) and the CVFs at 521 and 437 nm (Figure 3c) show the same behavior. As it is expected, if in the first potential cycle the oxidation potential exceeds +0.75 V, the absorbance at 303 nm and the fluorescence at 521 nm decrease due to the consumption and irreversible oxidation of OFL, while the fluorescence at 437 nm increases due to the generation of the OFL oxidation product. During the backward scan, when the potential of +0.75 V is reached, the CVA at 303 nm and CVF at 521 nm signals increase due to the diffusion of OFL from the bulk solution to the diffusion layer interrogated by the optical probes.

Furthermore, the CVF signal at 437 nm is diminished in intensity when the potential is lower than +0.75 V, owing to the diffusion of the oxidation product into the solution.

The behavior observed in the second potential scan is analogous to that observed during the initial scan. Lastly, the CVRs at the two characteristic bands of OFL (1397 and 1619 cm^–1^, Figure 3d) show that in the first potential scan OFL is not detected by SERS in the Raman spectra. The Raman intensity does not increase until the AuNPs are reduced in the second cathodic scan, as is observed in Figure S4b (orange lines). The Raman signal at 570 cm^–1^ demonstrates the oxidation/reduction of AuNPs, Figure S4b. The Raman signal at 1397 cm^–1^ demonstrates that Raman amplification did not occur when the experiment started toward reduction. The AuNPs must first be oxidized and then reduced to observe the corresponding Raman spectrum, suggesting that the reconstruction of the NPs surface plays a key role in the activation of the SERS phenomenon.

From the results, it can be concluded that the new device allowed us to obtain four independent signals in a single experiment, helping us to understand the chemical processes taking place during an electrochemical process, with all the signals being correlated with each other, providing a full picture of the electrode process. While the UV/vis absorption and PL optical signals show changes throughout the redox process of OFL, the Raman signal demonstrates that an activation of the SERS substrate is needed without prior oxidation and subsequent reduction of the AuNPs, no substrate capable of amplifying the Raman signal is created, and therefore no OFL spectrum is observed up to that point. The oxidation/reduction of AuNPs in this case illustrates that SERS signals are influenced not only by the size and shape of the particles but also by their surface condition, which can facilitate the chemical mechanism of enhancing the Raman signal.

## Conclusions

The coupling of electrochemistry with three spectroscopic techniques (UV/vis absorption, PL, and Raman) provides a comprehensive understanding of all the chemical processes involved in the electrode process. This is a major asset, offering a starting point for the analytical procedures used to identify and quantify the molecules of interest in multiple domains.

Raman analysis of OFL has demonstrated that when the experiment begins with a reduction step, no amplification of the Raman signal occurs. In other words, the initial AuNPs do not act as a SERS substrate, because the OFL Raman spectrum is not defined. However, when AuNPs are first oxidized and then reduced, the corresponding Raman spectrum emerges, demonstrating that the reconstruction of the AuNP surface plays a key role in the activation of the SERS phenomenon. This reconstruction suggests a new experimental pathway for the detection of this family of antibiotics using SERS.

## Supplementary Material



## Notes

Design and fabrication of the device for UV–vis absorption/PL/Raman SEC. Experimental Section: Instrumentation. Reagents and Materials. Synthesis of AuNPs. Electrode modification. Results and discussion: Ru­(bpy)_3_^2+^ spectra evolution during a UV/vis absorption, PL and Raman SEC experiment. Comparison of spectra and voltaRamangrams of Ru­(bpy)_3_^2+^ and ofloxacin during a UV/vis absorption, PL and Raman SEC experiment. Evolution of ofloxacin spectra during a UV/vis absorption, PL and Raman SEC experiment. (PDF)
