# Supplementary material for: Simultaneous UV/vis Absorption in Parallel Configuration, Photoluminescence and Raman Spectroelectrochemistry
Source: ACS Electrochem. 2025 Mar 22;1(6):997–1002. doi: 10.1021/acselectrochem.5c00038 (PMC12147439; doi:10.1021/acselectrochem.5c00038)
Supplement: Supplementary file 1 [file ec5c00038_si_001.pdf]

SUPPORTING INFORMATION

Simultaneous UV/vis Absorption in Parallel Configuration, Photoluminescence and Raman Spectroelectrochemistry

Fabiola Olmo[a], Martin Perez-Estebanez[a], Aranzazu Heras[a,*], Francisco Javier del Campo[b,c], Alvaro Colina[a,*]

[a] Department of Chemistry, Universidad de Burgos, Pza. Misael Bañuelos S/n, E-09001, Burgos, Spain.

[b] BCMaterials, Basque Center for Materials, Applications and Nanostructures, UPV/EHU Science Park, 48940 Leioa, Vizcaya, Spain.

[c] IKERBASQUE, Basque Foundation for Science, 48009 Bilbao, Spain.

*Corresponding Authors' e-mail: Aranzazu Heras (maheras@ubu.es), Alvaro Colina (acolina@ubu.es)

Table of contents

1. SEC CELL DETAILED DESCRIPTION S3

2. EXPERIMENTAL SECTION S5

2.1. Instrumentation S5

2.2. Reagents and Materials S6

2.3. Synthesis of gold nanoparticles (AuNPs) S7

2.4. Electrode modification S8

3. RESULTS AND DISCUSSION S9

3.1. $Ru(bpy)_3^{2+}$ spectra evolution during a UV/vis absorption, PL and Raman SEC experiment S9

3.2. Comparison of gold oxides evolution in the presence of $Ru(bpy)_3^{2+}$ and ofloxacin during a UV/vis absorption, PL and Raman SEC experiment S10

3.3. Ofloxacin spectra evolution during a UV/vis absorption, PL and Raman SEC experiment S11

4. REFERENCES S12

1. SEC CELL DETAILED DESCRIPTION

A real photo of the cell is displayed in Figure S1a. This cell consisted of three main parts, as shown in Figure 1B in the main text. Furthermore, each of the spectroscopic techniques is shown separately. Thus, the UV/vis absorption spectroscopy setup is depicted in Figure S1b and S1c, the PL assembly is shown in Figure S1d and the Raman setup corresponds to Figure S1e. The piece placed at the bottom (I) was created using a UV sensitive 405 nm resin (High Clear Resin, ANYCUBIC) with a resin 3D printer (Photon Mono SE, ANYCUBIC). This piece, measuring 65.0 × 40.0 × 8.0 mm, had four magnets at the corners and is designed to accommodate a screen-printed electrode in a recess. The second piece (II) was made of poly(methyl methacrylate) (PMMA) using a high-precision CO_2 laser cutting machine (PC 60/40KII), which was also created with four magnets located at the corners, facilitating the assembly with the lower part. The design of piece II (47.5 × 40.0 × 9.8 mm) was inspired by a similar cell reported in a previous article.[1] This piece was designed with two pillars to hold two 100 µm bare optical fibers (Ocean Optics), which will be used for UV/vis absorption measurements in parallel arrangement.[1] The fibers were fixed to two pillars of 2.6 mm diameter and 2.0 mm height. These pillars are attached to piece II (as can be seen in Figure S1c) by a rectangular recess of 2.0 mm wide, in such a way that the rest of the central part of the piece remains open. This allows the accessibility of the other two spectroscopic techniques to the electrode surface, facilitating the incorporation of the Raman probe and the fibers necessary for further fluorescence measurements.

The optical pathway, or the space between the two optical fibers fixed on the cell to carry out UV/vis absorption measurements in parallel configuration, was measured with ImageJ,[2] so the optical pathway is always known. In this work, the optical pathway for the $Ru(bpy)_3^{2+}$ experiments was 0.40 mm, whereas it was 0.68 mm for the ofloxacin (OFL) experiments. Two metal nuts on the left side were added to piece II, in order to incorporate the third piece (III), which has two magnets in the lower part, so it is easily fixed to the previous one. This third piece (21.0 × 26.0 × 30.5 mm) is also made of PMMA and has been designed with a diagonal recess that facilitates the incorporation of the optical fibers required to perform the PL experiments in a

side configuration. Nail polish was used to fix the optical fibers in pieces II and III, to ensure the stability of the optical signals in UV/vis absorption and PL experiments.

3D models of pieces (I, II, III) could be provided on demand. To obtain more information, contact the corresponding authors.

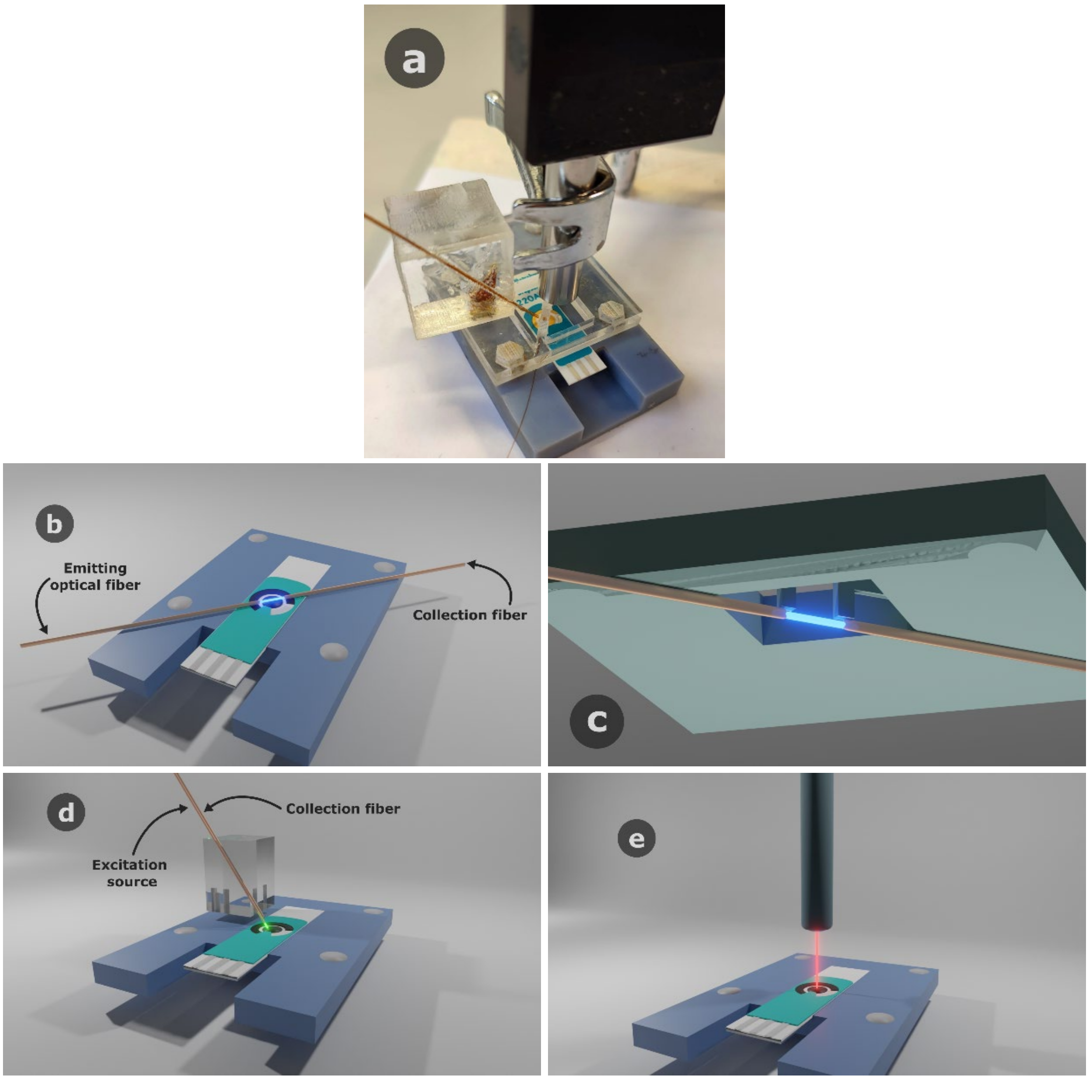

Figure S1. (a) Real photo of the SEC cell, (b) schematic image of the lower piece with the two optical fibers used to record the UV/vis absorption signal in a parallel configuration, (c) schematic image of the middle piece of the SEC cell with the two pillars to which the two optical fibers are attached, (d) schematic image of how the fibers are aligned on the piece III in order to collect the PL variation, (e) schematic image where the Raman probe is incorporated registering, in this case, the Raman signal.

2. EXPERIMENTAL SECTION

2.1. Instrumentation

SEC experiments were performed using three customized SPELEC instruments (Metrohm-DropSens) synchronized between them and controlled by DropView SPELEC software (Metrohm-DropSens). For *in situ* time-resolved UV/vis absorption SEC the customized SPELEC instrument includes a potentiostat, a halogen-deuterium light and a spectrometer. The customized PL-SPELEC instrument consists of a potentiostat, a spectrometer and a UV-LED with a maximum emission at 310 nm. In addition, the use of this LED allows the detection of the fluorescence of the two compounds under study, $Ru(bpy)_3^{2+}$ and OFL, without the interference from the excitation light. Finally, the SPELEC-Raman instrument includes a potentiostat, a spectrometer and a 785.18 nm laser source. A schematic representation of the integration of these devices is presented in Scheme S1. In addition, it is crucial to use filters to avoid saturating the CCD detectors of the spectrometers that record the absorbance and fluorescence signals with the light coming from the laser source. To register the UV/vis absorption signal, it is essential to use a band-pass filter (Ocean Optics) which permits the transmission of light with wavelengths within the range of 300-500 nm in the two cases studied in this work; thereby signal outside this spectral range are attenuated. The band-pass filter (LVF-UV-HL, Ocean Optics) is connected between a bare optical fiber (100 μm, Ocean Optics) and a commercial optical fiber (450 μm, QP450-1-XSR, Ocean Optics) that are connected to the spectrometer. In the case of PL measurements, a notch-filter (StopLine® Notch-filter, Semrock), which attenuates the signal at 785.18 cm^{-1}, is connected between two optical fibers, a bare optical fiber (100 μm, Ocean Optics) and other optical fiber (600 μm, QP600-025-VIS, Ocean Optics,). A notch-filter is required to minimize the light provided by the Raman probe, because without the filter, the spectrometer used in the PL measurements is saturated by Rayleigh scattering. The influence of PL on UV/vis absorption was controlled by proper positioning of the optical fibers, maximizing the distance between the PL emission fiber and the UV/vis collecting fiber. Moreover, PL could interfere with UV/vis absorption, but the emitted light collected in the absorption measurement is not significant with respect to the lamp light.

The setup could yield interference problems in the case of a PL system emitting at wavelengths longer than 785 nm. In this case, the position of the PL emitting fiber should be as far as possible from the Raman probe.

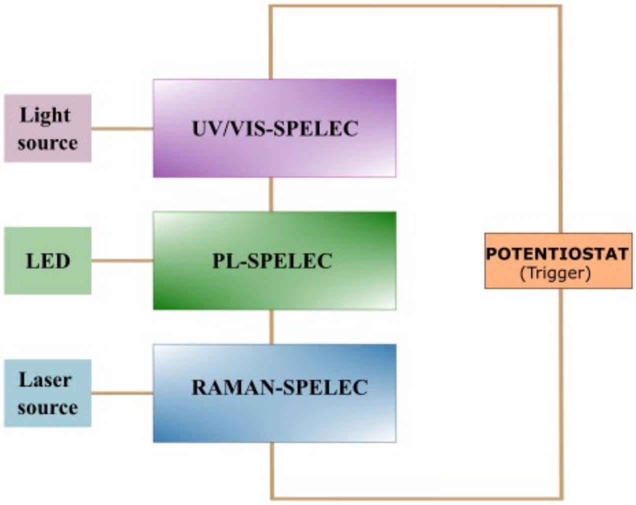

Scheme S1. Schematic representation of how different SPELEC instruments were connected. The potentiostat of the Raman-SPELEC instrument is the one that controls the electrochemical process. The three SPELEC instruments were connected by a trigger to ensure the synchronization of the three experiments.

A Zeiss GeminiSEM560 field-emission scanning electron microscope (FE-SEM) was used to obtain SEM images of the WE surface. An electron beam of 2 kV was used, with an in-lens secondary electrons detector.

2.2. Reagents and Materials

The designed SEC cell was validated using an aqueous solution of tris(2,2'-bipyridyl) ruthenium (II) chloride hexahydrate ($Ru(bpy)_3^{2+}$, Acros Organics) prepared in 0.1 M KNO_3 (Sigma-Aldrich), selected as the supporting electrolyte. Moreover, an ofloxacin (OFL, Sigma-Aldrich) aqueous solution was prepared in a 0.1 M Britton-Robinson (BR) medium, selected as the supporting electrolyte. The BR buffer solution contains boric acid (H_3BO_3, PANREAC), phosphoric acid (H_3PO_4, 85 %, PANREAC), acetic acid (HAc, VWR Chemicals) and sodium hydroxide (NaOH, Acros Organics). The pH of the BR buffer was adjusted to 4.25.

The following reagents are required for the preparation of the gold nanoparticle (AuNPs) dispersion: tetrachloroauric acid ($HAuCl_4$, Sigma-Aldrich), tri-sodium citrate dihydrate (Merck), and sodium hydroxide (NaOH, Acros Organics).

All reagents were used as provided without additional purification, and all solutions were prepared in high-quality ultrapure deionized water (18.2 MΩ cm resistivity at 25 °C, Milli-Q Direct 8, Millipore). All analyses were conducted at room temperature.

Modified gold screen-printed electrodes (Au-SPE, DRP-220AT, Metrohm-DropSens) to generate SERS substrates were used to carry out all the SEC experiments. The working electrode (WE), with a diameter of 4 mm, was modified with quasi-spherical 42 nm gold nanoparticles (AuNPs) to create a SERS substrate to amplify the Raman signal.

2.3. Synthesis of gold nanoparticles (AuNPs)

A well-known protocol has been used to synthesize AuNPs.[3–5] Quasi-spherical 42 nm AuNPs have been synthesized using a $2.5 \cdot 10^{-4}$ M of $HAuCl_4$ solution. This solution was heated to boiling point and then 0.375 mL of $3.4 \cdot 10^{-2}$ M tri-sodium citrate were added. The solution undergoes a change in color, becoming dark lilac 20 s after the addition of citrate, which indicates the beginning of the AuNP synthesis. Subsequently, the solution is boiled on the hotplate for 15 min, after which it is removed from the hotplate and cooled. Finally, a cleaning protocol is implemented for the synthesized AuNPs, consisting of the addition of NaOH to the dispersion, which causes the NPs precipitation. The supernatant is carefully removed to avoid removal of the AuNPs that precipitated to the bottom of the dispersion. This process is repeated two more times. A SEM image of clean AuNPs is shown in Figure S2.

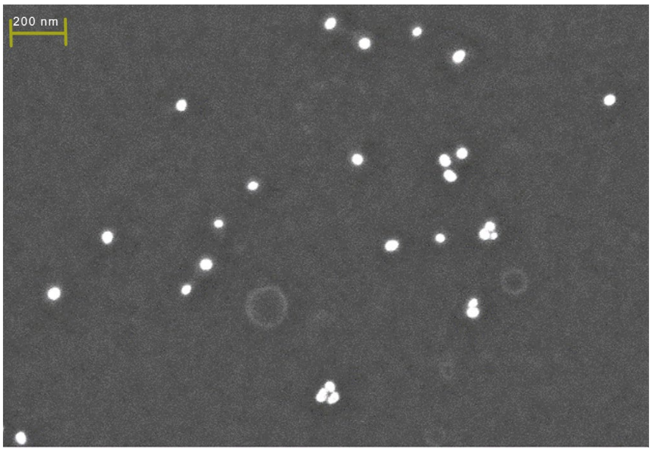

Figure S2. SEM image of synthesized and cleaned AuNPs.

2.4. Electrode modification

It is well known that Raman spectroscopy is characterized by intrinsically low sensitivity. To overcome this drawback, a SERS substrate is fabricated by modifying gold screen-printed electrodes (Au-SPE, DRP-220AT, Metrohm-DropSens). The working electrode (WE) of 4 mm diameter Au-SPEs is modified by the drop-casting method, whereby the aqueous solution of the quasi-spherical 42 nm AuNPs is deposited to create a SERS substrate that can amplify the Raman signal.

In this sense, 5 µL of the AuNPs dispersion are deposited on the WE-Au-SPE and allowed to dry until the solvent is completely evaporated. This process is repeated two more times. To accelerate the drying process of the 5 µL droplets, a nitrogen stream is applied.

3. RESULTS AND DISCUSSION

3.1. Ru(bpy)$_3^{2+}$ spectra evolution during a UV/vis absorption, PL and Raman SEC experiment

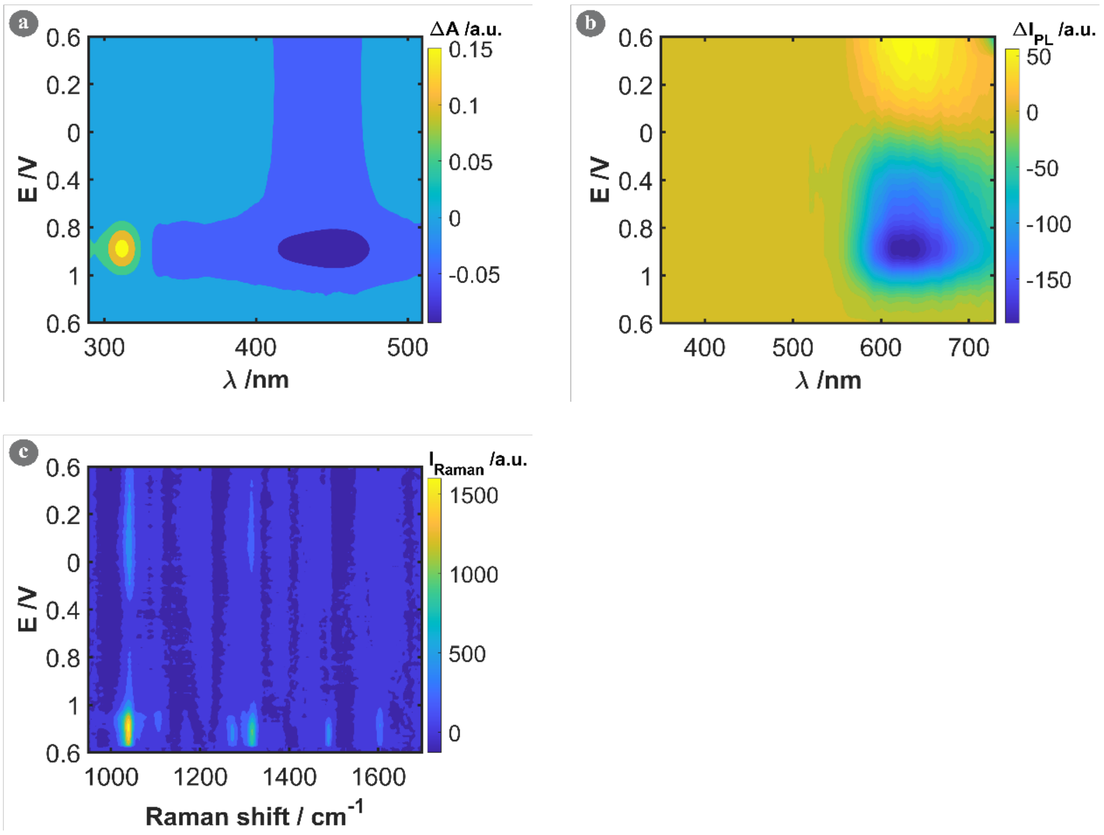

Figure S3. Spectra evolution of 10^{-3} M Ru(bpy)$_3^{2+}$ in 0.1 M KNO_3 between -0.10 V and +1.10 V at 0.02 Vs^{-1}, starting at +0.60 V in the anodic direction. (a) UV/vis absorption spectra, (b) PL spectra and (c) Raman spectra.

Figure S3 represents the contour plots for all spectroscopic techniques obtained during the experiment shown in Figure 2 in the main text: UV/vis absorption (Figure S3a), PL (Figure S3b) and Raman (Figure S3c). For UV/vis absorption spectroscopy, a reference spectrum at OCP was taken before the CV. Thus, a 0 in absorbance is defined as the initial state of the experiment. A similar strategy was taken for PL, since the variation of the emission with respect to the first spectra was plotted. For PL, 0 emission represents the initial state of the experiment.

Table S1. Band assignments of Raman spectrum for $Ru(bpy)_3^{2+}$ in 0.1 M KNO_3 during the SEC experiment, shown in Figure S3.

EC-SERS bands for $Ru(bpy)_3^{2+}$	Mode assignments[6]
1039	Ru-N stretching, ring breathing
1273	C-H wagging, C-N stretching
1318	2-2' C-C stretching, C-H wagging
1487	C-C stretching, C-H wagging
1560	C-C stretching, C-H wagging
1602	C-C stretching, C-H wagging

3.2. Comparison of gold oxides evolution in the presence of $Ru(bpy)_3^{2+}$ and ofloxacin during a UV/vis absorption, PL and Raman SEC experiment

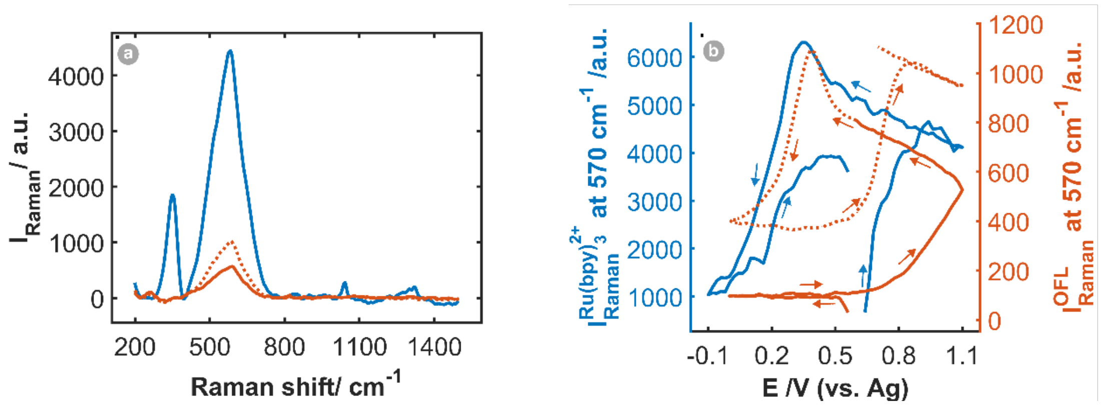

Figure S4. (a) Raman spectra at +1.10 V of $1\cdot10^{-3}$ M $Ru(bpy)_3^{2+}$ in 0.1 M KNO_3 (blue line) and of $5\cdot10^{-4}$ M OFL in BR pH = 4.25 (orange lines, solid line 1st cycle, dotted line 2nd cycle), (b) voltaRamangrams at 570 cm^{-1} of $1\cdot10^{-3}$ M $Ru(bpy)_3^{2+}$ in 0.1 M KNO_3 (blue line) and of $5\cdot10^{-4}$ M OFL in BR pH = 4.25 (orange lines, solid line 1st cycle, dotted line 2nd cycle)

Figure S4a represents the Raman spectra at potential vertex for the experiments shown in Figures 2 and 3 in the main text. As can be seen, a noticeable band centered around 570 cm^{-1} is observed in these conditions. Figure S4b represents the evolution of these bands in their corresponding experiments, revealing its evolution at potentials above +0.80 V, and rapidly decreases at potentials lower than +0.40 V. These potentials coincide with the oxidation and reduction potential of AuNPs (O1, R2 in Figure 2 and Figure 3 in the main text), respectively, which reveals that the band is related to gold oxides. Noticeably, at the potentials where this Raman band

decreases, it is observed the Raman enhancement of $Ru(bpy)_3^{2+}$ and OFL, confirming that the reduction of oxides leads to the activation of the SERS properties of AuNPs.

3.3. Ofloxacin spectra evolution during a UV/vis absorption, PL and Raman SEC experiment

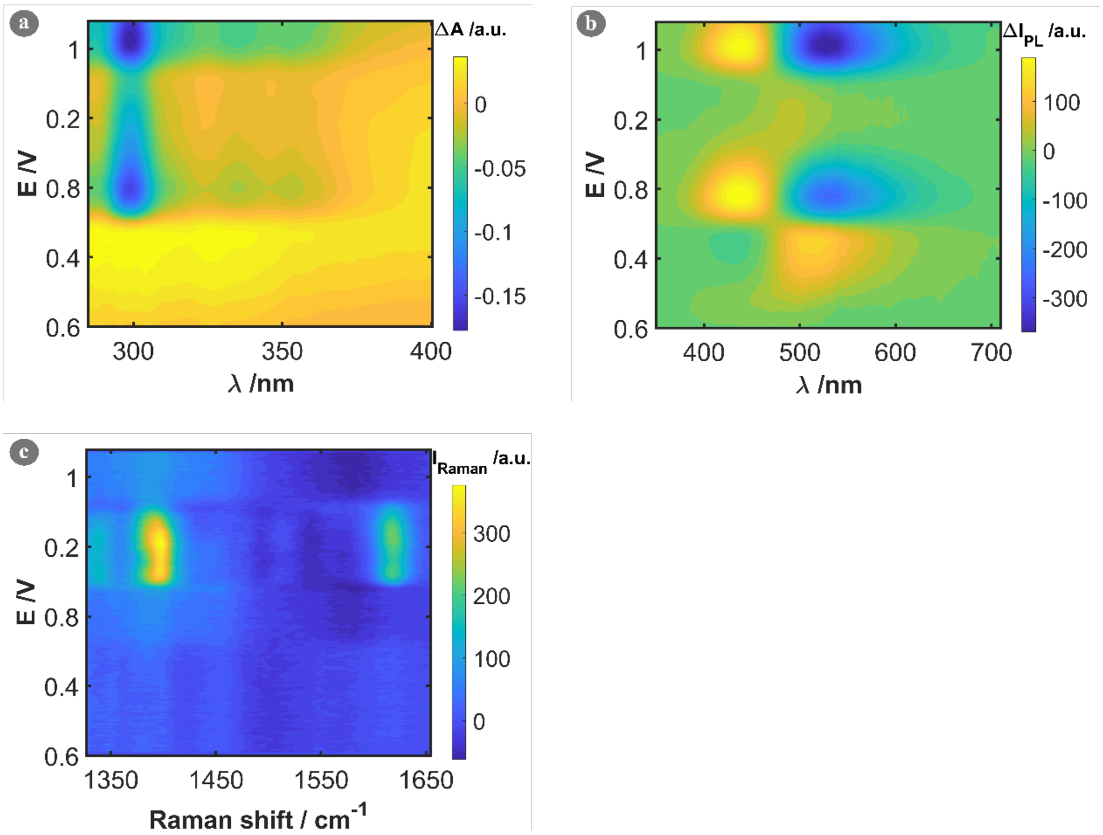

Figure S5. Spectra evolution of $5 \cdot 10^{-4}$ M OFL in BR pH = 4.25 at 0.02 $V \cdot s^{-1}$ between 0.00 V and +1.10 V, starting at +0.60 V in the cathodic direction. 2 cycles are plotted. (a) UV/vis absorption spectra, (b) PL spectra and (c) Raman spectra.

Figure S5 represents the contour plots for all spectroscopic techniques obtained during the experiment shown in Figure 3 in the main text: UV/vis absorption (Figure S5a), PL (Figure S5b) and Raman (Figure S5c). For UV/vis absorption spectroscopy, a reference spectrum was taken before the CV. Thus, a 0 in absorbance is defined as the initial state of the experiment. A similar strategy was taken for PL, since the variation of the emission with respect to the first spectra was plotted. For PL, 0 emission represents the initial state of the experiment.

4. REFERENCES

(1) Olmo, F.; Colina, A.; Heras, A. Determination of Ofloxacin in Urine Using UV/vis Absorption Spectroelectrochemistry. *Microchem. J.* **2024**, *198*, 110186. https://doi.org/10.1016/j.microc.2024.110186.

(2) Schneider, C. A.; Rasband, W. S.; Eliceiri, K. W. NIH Image to ImageJ: 25 Years of Image Analysis. *Nat. Methods* **2012**, *9* (7), 671–675. https://doi.org/10.1038/nmeth.2089.

(3) Verma, H. N.; Singh, P.; Chavan, R. M. Gold Nanoparticle: Synthesis and Characterization. *Vet. World* **2014**, *7* (2), 72–77. https://doi.org/10.14202/vetworld.2014.72-77.

(4) Amir Tabrizi, Fatma Ayhan, H. A. Gold Nanoparticle Synthesis and Characterisation. *Hacettepe J. Biol. Chem.* **2009**, *37* (3), 217–226.

(5) Shiraishi, Y.; Tanaka, H.; Sakamoto, H.; Ichikawa, S.; Hirai, T. Photoreductive Synthesis of Monodispersed Au Nanoparticles with Citric Acid as Reductant and Surface Stabilizing Reagent. *RSC Adv.* **2017**, *7* (11), 6187–6192. https://doi.org/10.1039/C6RA27771C.

(6) Silverstein, D. W.; Milojevich, C. B.; Camden, J. P.; Jensen, L. Investigation of Linear and Nonlinear Raman Scattering for Isotopologues of $Ru(bpy)_3^{2+}$. *J. Phys. Chem. C* **2013**, *117* (40), 20855–20866. https://doi.org/10.1021/jp4070505.
